# Structural plasticity of spines at giant mossy fiber synapses

**DOI:** 10.3389/fncir.2012.00103

**Published:** 2012-12-18

**Authors:** Shanting Zhao, Daniel Studer, Xuejun Chai, Werner Graber, Nils Brose, Sigrun Nestel, Christina Young, E. Patricia Rodriguez, Kurt Saetzler, Michael Frotscher

**Affiliations:** ^1^Center for Molecular Neurobiology Hamburg, Institute for Structural NeurobiologyHamburg, Germany; ^2^Institute of Anatomy, University of BernBern, Switzerland; ^3^Department of Molecular Neurobiology, Max Planck Institute for Experimental MedicineGöttingen, Germany; ^4^Department of Neuroanatomy, Institute of Anatomy and Cell Biology, Albert Ludwigs University FreiburgFreiburg, Germany; ^5^School of Biomedical Science, University of UlsterColeraine, UK

**Keywords:** synaptic ultrastructure, high-pressure freezing, mossy fiber LTP, dendritic spine, actin cytoskeleton, dentate gyrus, granule cells

## Abstract

The granule cells of the dentate gyrus give rise to thin unmyelinated axons, the mossy fibers. They form giant presynaptic boutons impinging on large complex spines on the proximal dendritic portions of hilar mossy cells and CA3 pyramidal neurons. While these anatomical characteristics have been known for some time, it remained unclear whether functional changes at mossy fiber synapses such as long-term potentiation (LTP) are associated with structural changes. Since subtle structural changes may escape a fine-structural analysis when the tissue is fixed by using aldehydes and is dehydrated in ethanol, rapid high-pressure freezing (HPF) of the tissue was applied. Slice cultures of hippocampus were prepared and incubated *in vitro* for 2 weeks. Then, chemical LTP (cLTP) was induced by the application of 25 mM tetraethylammonium (TEA) for 10 min. Whole-cell patch-clamp recordings from CA3 pyramidal neurons revealed a highly significant potentiation of mossy fiber synapses when compared to control conditions before the application of TEA. Next, the slice cultures were subjected to HPF, cryosubstitution, and embedding in Epon for a fine-structural analysis. When compared to control tissue, we noticed a significant decrease of synaptic vesicles in mossy fiber boutons and a concomitant increase in the length of the presynaptic membrane. On the postsynaptic side, we observed the formation of small, finger-like protrusions, emanating from the large complex spines. These short protrusions gave rise to active zones that were shorter than those normally found on the thorny excrescences. However, the total number of active zones was significantly increased. Of note, none of these cLTP-induced structural changes was observed in slice cultures from Munc13-1 deficient mouse mutants showing severely impaired vesicle priming and docking. In conclusion, application of HPF allowed us to monitor cLTP-induced structural reorganization of mossy fiber synapses.

## Introduction

The giant synapses formed by hippocampal mossy fibers, the axons of dentate gyrus granule cells, have attracted researchers soon after the introduction of electron microscopy (EM) to the study of the nervous system (Blackstad and Kjaerheim, 1961; Hamlyn, 1962). Via the mossy fiber synapses, the granule cells transmit afferent, multisensory input from the entorhinal cortex to the hippocampus proper. Intuitively, one expects that the granule cells are bipolar neurons in order to serve this function. Indeed, they send their dendrites into the dentate molecular layer where the afferents from the entorhinal cortex are known to terminate in a laminated fashion. This input side of the granule cells is clearly segregated from their output side, represented by the mossy fibers projecting to the hilus and farther to hippocampal region CA3. The layer of granule cell somata is forming a “border” between the two sides. In fact, in the normal dentate gyrus, granule cells rarely show basal dendrites extending into the hilus so that they could become a target of other granule cell axons. It has been shown that hilar ectopic granule cells receive many more excitatory synapses and show epileptiform burst activity unlike normal granule cells in the dentate granule cell layer (Dashtipour et al., 2001; Scharfman et al., 2007). Hence, granule cell ectopia resulting from an aberrant migration of the granule cells during development has been assumed to increase the susceptibility to developing limbic seizures and epilepsy in adulthood (Koyama et al., 2012). While this may be the case, granule cell ectopia may also be a secondary effect that can be provoked in normal mature animals by unilateral injection of the glutamate receptor agonist kainate into the hippocampus (Bouilleret et al., 1999; Heinrich et al., 2006). Application of kainate renders the animals epileptic, and they then develop a prominent, secondary granule cell ectopia, called granule cell dispersion. For the purpose of the present study it may be sufficient to point out that the information flow is mainly unidirectional in the normal dentate gyrus conveying the multisensory entorhinal input to the hippocampus via essentially two synapses, the synapses formed by the entorhinal fibers on the granule cell dendrites and the synapses that the mossy fibers establish with hilar neurons and CA3 pyramidal cells, respectively.

The input from the entorhinal cortex is not the only input to the granule cell dendrites. The commissural/associational fibers terminating on proximal granule cell dendrites are derived from distant hilar mossy cells that transmit input from other segments (lamellae) of the longitudinal axis of the hippocampus. The mossy fibers, in turn, play an important role in the activation of local (intralamellar) mossy cells. Other target cells of granule cell axons are the various types of GABAergic interneuron (Frotscher, 1985, 1989; Acsády et al., 1998) that by recurrent and feed-forward inhibition, respectively, keep the balance between excitation and inhibition in the dentate-hippocampal network.

In this review, we will first summarize what is known about the mossy fibers and their characteristic synaptic specializations on hippocampal CA3 pyramidal neurons. We will make an attempt to understand the functional significance of the unique structural components of mossy fiber synapses, and we will study the structural plasticity of mossy fiber synapses using high-pressure freezing (HPF), an approach that allowed us to overcome some of the disadvantages of conventional aldehyde fixation for EM.

## The specialized mossy fiber synapse

What is so special about the giant mossy fiber synapse? The thin unmyelinated granule cell axon originates at the basal pole of the granule cell soma and travels through the hilus toward hippocampal region CA3. In its course, it gives rise to regularly spaced boutons *en passage* for the contact with various types of hilar neuron. Toward CA3, the mossy fibers form a dense fiber bundle that occupies stratum lucidum. Depending on the genetic background, some mossy fibers also run through the layer of pyramidal cells (intrapyramidal mossy fibers) and underneath the pyramidal layer (infrapyramidal bundle, Schwegler et al., 1981). The mossy fiber projection ends abruptly at the border to CA2/CA1. Thus, the CA1 pyramidal cells do not get direct granule cell input, suggesting that the information flow from the entorhinal cortex via the granule cells requires just another processing before it is allowed to enter the CA1 region (there is, however, a direct entorhinal input to the CA1 pyramidal cells). One is tempted to speculate that at the level of the CA3 pyramidal cells there is a comparative evaluation of the individual granule cell projections, likely by abundant connections among CA3 pyramidal cells, aimed at providing the CA1 neurons with a more complete pattern of the lamellar granule cell input from the dentate gyrus, whereby contextual information is provided by direct projections from the entorhinal cortex to the hippocampus proper (Lisman, 1999).

## What does the particular fine structure of the mossy fiber synapse tell us about its functional role in the dentate—hippocampal circuit?

**Figure 1:**
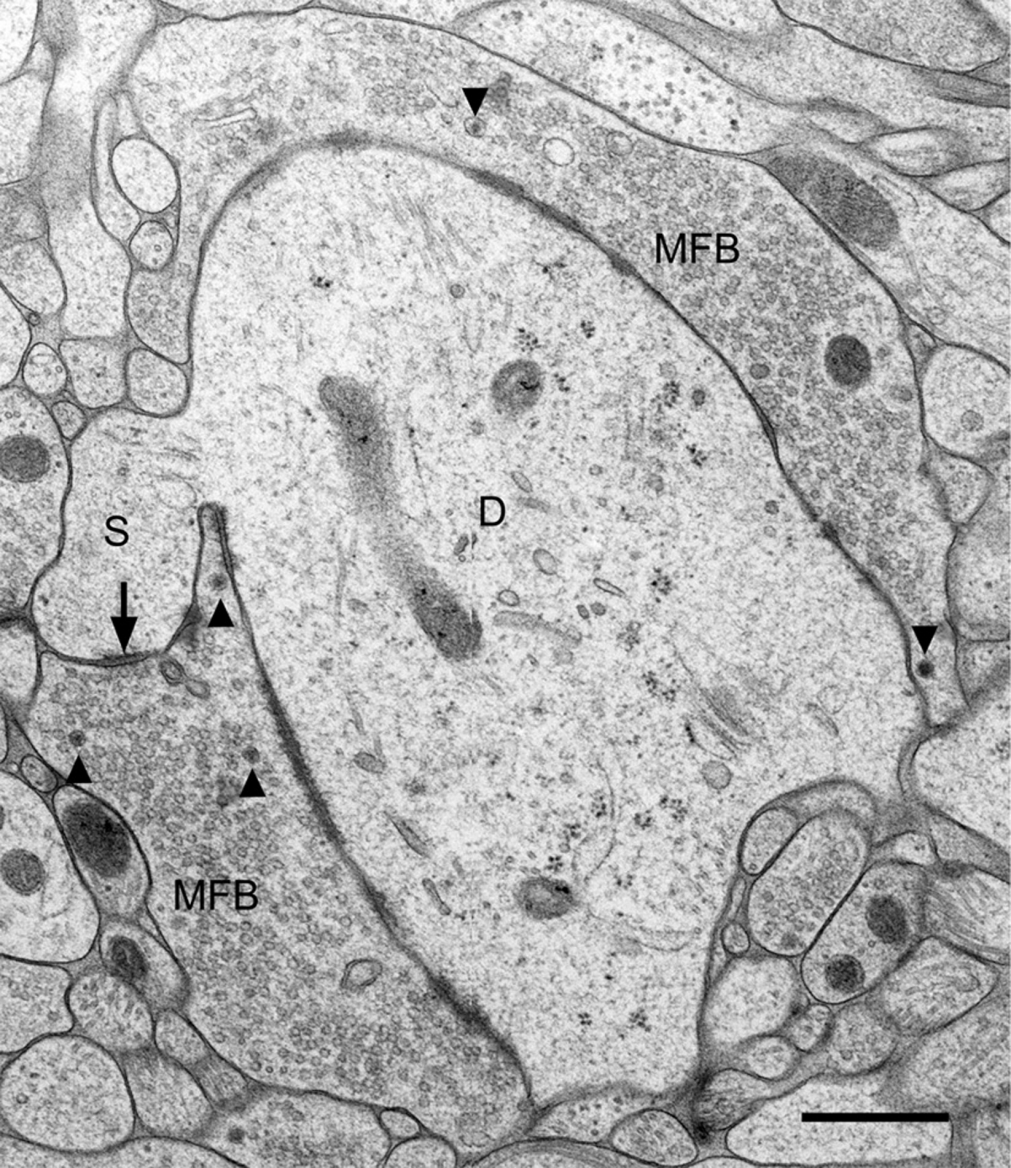
**Fine structure of a large mossy fiber bouton (MFB) and its postsynaptic element, a proximal dendrite (D) of a CA3 pyramidal cell.** The large complex spine (S) establishes synaptic contact (arrow) with the mossy fiber bouton. Arrowheads point to dense-core vesicles. Slice culture of hippocampus following high-pressure freezing, cryosubstitution, and embedding in Epon. Scale bar: 0.5 μm.

The location of mossy fiber boutons on proximal dendritic portions, i.e., close to the site of action potential generation, the enormous bouton size (2–5 μm in diameter), the abundance of clear synaptic vesicles (approximately 25,000) intermingled by dense–core vesicles, and the large number of release sites (up to 45 release sites, see Chicurel and Harris, 1992; Rollenhagen et al., 2007) strongly suggests that this synapse plays an important role in the information flow within the “trisynaptic pathway” (Andersen et al., 1971) connecting the entorhinal cortex with the hippocampus proper via the granule cells. It is likely that depolarization of the presynaptic mossy fiber axon leading to Ca^2+^ influx into the mossy fiber bouton will result in the simultaneous activation of several of the 45 contact zones, thereby increasing the reliability of transmission at the mossy fiber synapse. The particular fine structure of a mossy fiber synapse is illustrated in Figure 1. Here, the tissue was not fixed by using aldehydes and dehydrated in ascending series of ethanol. The tissue (slice culture of mouse hippocampus) was shock-frozen under high-pressure in less than a second (Zhao et al., 2012), thereby preserving the characteristic ultrastructural details of this synapse and avoiding ethanol-induced shrinkage of tissue components. While the fine-structural characteristics of mossy fiber synapses have been known for more than 50 years (Blackstad and Kjaerheim, 1961; Hamlyn, 1962) and were all described by studying tissue fixed in aldehyde solution and dehydrated in ethanol, the study of rapid, subtle changes at mossy fiber synapses, in particular at the giant postsynaptic spines, likely requires a rapid “fixation” procedure such as HPF. We will show in fact, that the mossy fiber synapse is a highly dynamic structure and that functional synaptic plasticity is associated with structural changes in the presynaptic bouton as well as the postsynaptic spine compartment.

## Structural changes associated with functional plasticity at mossy fiber synapses

A variety of recent studies have shown that synaptic plasticity such as long-term potentiation (LTP) is associated with structural changes at synapses, particularly in the number and size of dendritic spines (Engert and Bonhoeffer, 1999; Matsuzaki et al., 2001; Yuste and Bonhoeffer, 2001; Yang et al., 2008). Are functional changes at mossy fiber synapses associated with structural changes? With the concept that induction of chemical LTP (cLTP) results in the stimulation of virtually all potentiatable mossy fiber synapses in contrast to electrical stimulation that activates an indeterminate number of synapses (Hosokawa et al., 1995), we induced cLTP by the application of tetraethylammonium (TEA; 25 mM) to hippocampal slice cultures for 10 min. Following washout, we recorded from CA3 pyramidal cells using the patch-clamp technique in the whole-cell mode. As shown previously (Suzuki and Okada, 2009; Zhao et al., 2012), there was a strong potentiation of excitatory postsynaptic potentials (EPSPs) 10–15 min after washout. Next, slice cultures exposed to TEA for 10 min were subjected to HPF and embedded for EM (Zhao et al., 2012). Control slice cultures were treated exactly the same way except that they were not exposed to TEA. In addition, we used slice cultures from Munc13-1 mouse mutants in which synaptic vesicle priming and docking is compromised (Augustin et al., 1999).

We measured the following parameters of mossy fiber synapses in random sections of control cultures and cultures treated with TEA: Number of clear synaptic vesicles (we also made an attempt to quantify dense-core vesicles but their number was too low for a statistical analysis), area and perimeter of mossy fiber boutons, number and area of postsynaptic spines, number and length of synaptic contacts (active zones or release sites).

Electron microscopic analysis of high-pressure frozen slice cultures—controls and TEA-exposed cultures—revealed an exceptional preservation of fine-structural detail (Figure 1).

Not only were the membranes of mossy fiber boutons and of postsynaptic spines and dendrites very smooth and crisp, also intracellular organelles such as microtubules, mitochondria, endoplasmic reticulum, synaptic vesicles, and in particular active zones were clearly identifiable. Postsynaptic densities extended fine fibrillar structures, likely protein accumulations, from the subsynaptic web into the spine cytoplasm. Occasionally, vesicles showed small extensions tethering them to other vesicles or to the presynaptic membrane specialization.

The excellent tissue preservation allowed for a thorough quantitative analysis of the various tissue components and for a comparison with tissue fixed conventionally by transcardial perfusion with aldehyde solutions (see Zhao et al., 2012). When comparing control slice cultures with slice cultures exposed to TEA for 10 min, we noticed a highly significant reduction in the number of clear synaptic vesicles/μm^2^ mossy fiber bouton area (control: 183 ± 76 SD; TEA: 104 ± 43 SD; *p* = 0.000015, Zhao et al., 2012). This decrease in vesicle number was associated with an increase in the ratio of bouton perimeter/bouton area in the TEA-treated cultures (control: 3.85 ± 1.15 SD; TEA: 6.05 ± 1.52 SD; *p* = 0.00000018, Zhao et al., 2012). Since no significant differences between controls and stimulated cultures were found in a comparison of mossy fiber bouton area, we interpret these findings as resulting from an increased length and a more convoluted course of the presynaptic bouton membrane following the fusion of many synaptic vesicles.

**Figure 2:**
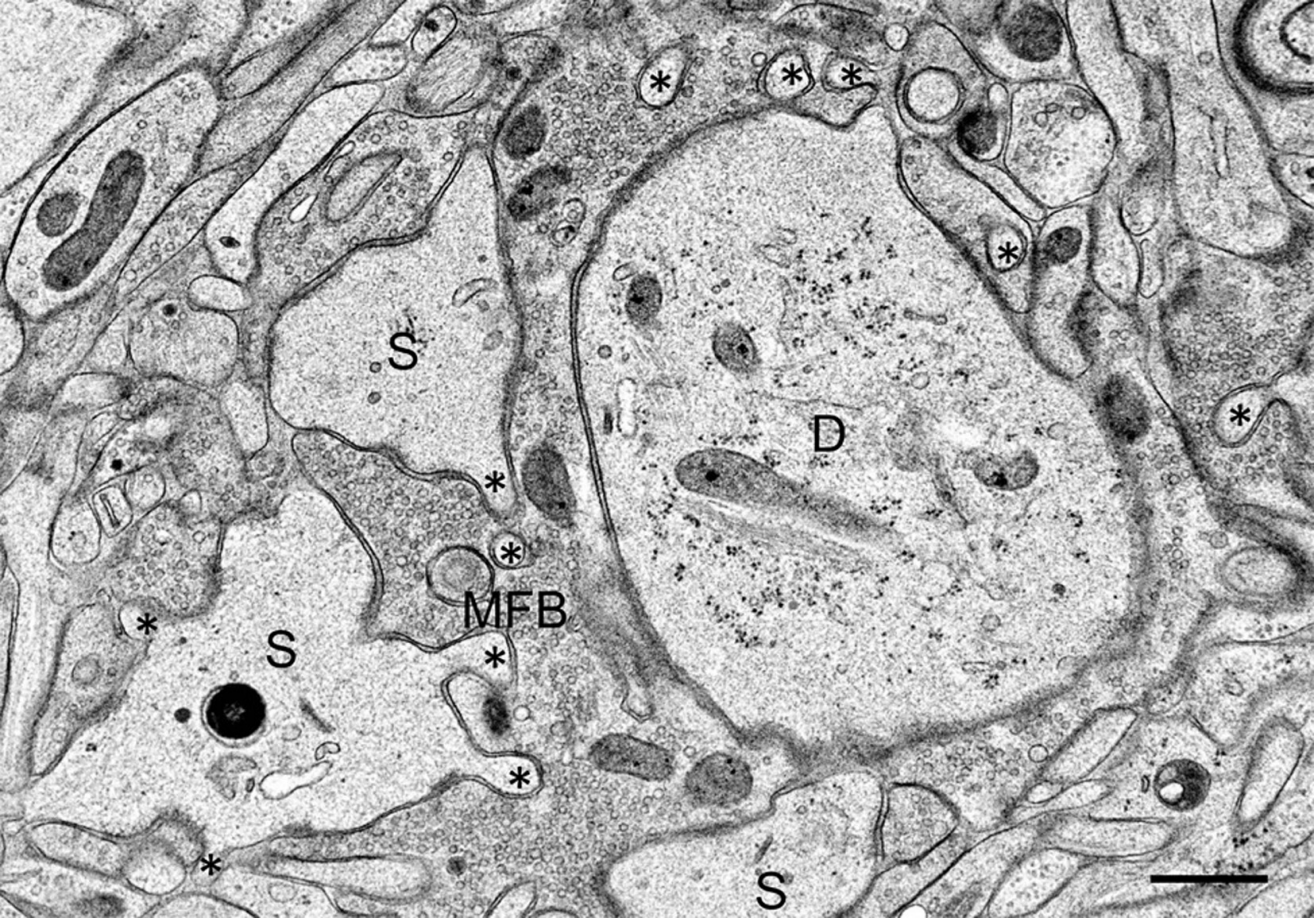
**Mossy fiber bouton (MFB) and its postsynaptic spines (S) in a hippocampal slice culture exposed to TEA for 10 min prior to high-pressure freezing.** Note finger-shaped small extensions (asterisks) emerging from the large complex spines. D, proximal CA3 pyramidal cell dendrite. Scale bar: 0.5 μm.

On the postsynaptic side, we observed in the TEA-exposed cultures numerous finger-like protrusions extending from the large complex spines or *excrescences* into the presynaptic bouton (Figure 2). Accordingly, quantitative analysis revealed a highly significant increase in the number of spine profiles/μm^2^ bouton area in the TEA-treated cultures when compared to controls (control: 0.42 ± 0.54 SD; TEA: 4.32 ± 2.19 SD; *p* = 1.569 × 10^−10^, Zhao et al., 2012). Similarly, spine area/mossy fiber bouton area was increased in the potentiated cultures (control: 0.134 ± 0.043 SD; TEA: 0.227 ± 0.078 SD; *p* = 0.0173, Zhao et al., 2012). Next, we wanted to know whether this *de novo* formation of spines was accompanied by an increase in the number of active zones. Indeed, we observed a significant increase in the number of synaptic contacts/μm^2^ bouton area in the potentiated cultures (control: 2.64 ± 1.21 SD; TEA: 3.63 ± 1.42 SD; *p* = 0.00882). However, the mean length of active zones was decreased when compared to control cultures (control: 221.8 nm ± 75.04 nm SD; TEA: 168.69 nm ± 28.63 nm SD; *p* = 0.04031, Zhao et al., 2012), suggesting that in the potentiated cultures many synapses were still immature.

We regarded it as an important result and additional control for the present experiments that none of the changes in synaptic structure found in the potentiated cultures was observed in potentiated slice cultures from mutant mice deficient in Munc13-1 (Zhao et al., 2012). Munc13-1 is known to be important for the fusion competence of synaptic vesicles (Augustin et al., 1999), and mutant mice deficient in Munc13-1 show severely compromised vesicle docking and fusion. Together, the present findings suggest that cLTP-induced by a 10-min exposure to TEA resulted in the fusion of many vesicles that, in turn, increased the length of the presynaptic mossy fiber bouton membrane. These changes were accompanied on the postsynaptic side by the formation of new spines and synaptic contacts. In Munc13-1 mutants, in which vesicle priming, docking and fusion are compromised, these changes were not observed. We hypothesize that under *in vivo* conditions, when only a limited number of granule cells are activated by specific input from the entorhinal cortex, their respective individual mossy fiber synapses are strengthened by closely related pre- and postsynaptic mechanisms eventually leading to additional spines and synaptic contacts.

## What mechanisms might underlie the cLTP-induced remodeling of mossy fiber synapses?

In the potentiated cultures we observed an increase in the number of spines and active zones. One way to explain the extension of the finger-shaped protrusions from the postsynaptic complex spines is by assuming that this enlargement of the postsynaptic surface occurred in response to presynaptic membrane growth resulting from the fusion of numerous vesicles (Zhao et al., 2012). Pre- and postsynaptic membranes of mossy fiber synapses are attached to each other by abundant synaptic contacts that do not only represent release sites but sites of strong mechanical contact (Gray and Whittaker, 1962), likely by virtue of numerous cell adhesion molecules at synaptic sites (Washbourne et al., 2004; Hortsch and Umemori, 2009). Alternatively, there might be active growth of postsynaptic spines and synaptic contacts. Indeed, our studies have shown that both spines and active zones were smaller than in controls, suggesting that these synaptic structures were still growing after the 10-min stimulation period. It has been shown that LTP induction in CA1 is associated with *de novo* formation and growth of dendritic spines, respectively (Engert and Bonhoeffer, 1999; Yuste and Bonhoeffer, 2001; Matsuzaki et al., 2004; Yang et al., 2008). Growth of spines is accompanied by an increase in the number of functional glutamate receptors (Matsuzaki et al., 2001; Noguchi et al., 2005; Béïque et al., 2006; Zito et al., 2009; Kasai et al., 2010) that are likely to contribute to LTP and memory consolidation.

Growth of spines is associated with remodeling of the actin cytoskeleton, which is particularly enriched in dendritic spines (Matus, 2000). Indeed, a variety of studies have provided evidence for changes in actin filaments to be linked to changes in spine shape under various experimental conditions (Fischer et al., 1998, 2000; Matus, 2000; Star et al., 2002; Fukazawa et al., 2003; Okamoto et al., 2004; Hotulainen et al., 2009; Hotulainen and Hoogenraad, 2010). Remodeling of spine structure in synaptic plasticity was found to involve changes in the equilibrium between F-actin and G-actin by actin polymerizing/depolymerizing molecules. Thus, opposing shifts in the relative amounts of F-actin and G-actin have been observed in bidirectional synaptic plasticity: LTP shifted the equilibrium toward F-actin, associated with an increase in the size of spines on CA1 pyramidal neurons; long-term depression (LTD) shifted the equilibrium toward G-actin and resulted in smaller spines (Okamoto et al., 2004). Consistent with increased actin polymerization and an increase in spine size, phosphorylated cofilin (p-cofilin) was found increased in LTP in hippocampal region CA1 (Chen et al., 2007; Rex et al., 2009). Cofilin is an actin-depolymerizing protein and involved in cytoskeletal remodeling associated with changes in cell shape (Bamburg, 1999). Phosphorylation of cofilin at serine3 renders it unable to depolymerize F-actin (Arber et al., 1998; Yang et al., 1998), thereby stabilizing the actin cytoskeleton. In preliminary studies using high-pressure frozen material and immunogold staining for p-cofilin following embedding in Lowicryl HM20, we observed labeling specifically at synaptic sites (Zhao et al., unpublished observations). Similar synaptic location of cofilin has been reported previously using an antibody against the N-terminal region of human cofilin (Racz and Weinberg, 2006). Future studies need to determine to what extent changes in the actin cytoskeleton and in the phosphorylation of cofilin are involved in the cLTP-induced structural plasticity of spines postsynaptic to giant mossy fiber boutons.

## Discussion and outlook

The results presented here provide evidence for functional changes such as synaptic potentiation at mossy fiber synapses to be associated with structural changes, in particular a *de novo* formation of spines and synaptic contacts. Thus, our results confirm and extend previous studies on LTP-induced structural changes in spines of CA1 pyramidal neurons. Our findings in mossy fiber synapses imply that a selective activation of single granule cells, for instance by strong input from the entorhinal cortex, strengthens their respective synapses on hilar mossy cells and CA3 pyramidal neurons by the formation of additional synaptic contacts and spines postsynaptic to these particular mossy fiber boutons. As a result, the respective mossy fiber synapses will become more convoluted and complex. Indeed, previous studies have shown that long-term experience resulted in rearrangements of presynaptic mossy fiber boutons (Galimberti et al., 2006, 2010; Rekart et al., 2007; Routtenberg, 2010). At a systemic level, these more complex mossy fiber synapses may reinforce transmission in the trisynaptic pathway in the particular lamella of the activated granule cell(s). Thus, by virtue of the preferentially transverse course of the mossy fibers (transverse to the longitudinal axis of the hippocampus, Blackstad et al., 1970) individual lamellae will be activated; however, by the simultaneous activation of the mossy cells that project along the longitudinal axis of the hippocampus, the impulse will not remain restricted to the lamella but will be propagated to distant septo-temporal segments of the hippocampus.

What signaling pathways and molecular mechanisms might underlie the *de novo* formation of spines and synaptic contacts? Previous studies in CA1 have not only shown that LTP induces spine formation (Engert and Bonhoeffer, 1999; Yuste and Bonhoeffer, 2001; Matsuzaki et al., 2004; Yang et al., 2008), but have also provided evidence for an involvement of brain-derived neurotrophic factor (BDNF) in LTP (Korte et al., 1995; Lu et al., 2008). BDNF is particularly enriched in hippocampal mossy fibers (Danzer and McNamara, 2004), and we have recently shown that it is localized to dense-core vesicles of mossy fiber boutons (Dieni et al., 2012). One is tempted to speculate that the structural plasticity of mossy fiber synapses described here is regulated by BDNF released from dense-core vesicles in an activity-dependent manner. It has been shown recently that phosphorylation of TrkB, the cognate receptor for BDNF, by the proline-directed serine/threonine kinase Cdk5 is required for activity-dependent plasticity of dendritic spines and spatial memory (Lai et al., 2012). Future studies in slice cultures from BDNF-deficient mutant mice will show to what extent the structural plasticity of mossy fiber synapses described here is affected by a BDNF deficiency.

Electron microscopy does not allow one to study the time course of structural changes at mossy fiber synapses. How rapid are these changes in response to mossy fiber stimulation?

In order to address this issue, we have recently begun to patch-clamp CA3 pyramidal cells and hilar mossy cells, respectively, and have filled them with the fluorescent marker Alexa 594-dextran (red). Presynaptic mossy fiber axons were stained by the extracellular application of Alexa 588-dextran (green). This experimental design will allow us to monitor pre- and postsynaptic changes at identified mossy fiber synapses using real-time microscopy (Frotscher et al., 2007). Together with the use of slice cultures from BDNF-deficient mice and from other mutants with modifications of candidate molecules, this approach will allow us to obtain further insight into the mechanisms underlying the structural plasticity of mossy fiber synapses.

### Conflict of interest statement

The authors declare that the research was conducted in the absence of any commercial or financial relationships that could be construed as a potential conflict of interest.
